# Study on Di-Phase Membrane Device with DZ272(DDD) for Purification Behavior of Divalent Cobalt Ions in Slops

**DOI:** 10.3390/toxics10090546

**Published:** 2022-09-19

**Authors:** Liang Pei, Liying Sun

**Affiliations:** 1 National Engineering Technology Research Center for Desert-Oasis Ecological Construction, Xinjiang Institute of Ecology and Geography, Chinese Academy of Sciences, Urumqi 830011, China; 2 Institute of Geographic Sciences and Natural Resources Research, Chinese Academy of Sciences, Beijing 100101, China; 3 Xinjiang Key Laboratory of Environmental Pollution and Bioremediation, Xinjiang Institute of Ecology and Geography, Chinese Academy of Sciences, Urumqi 830011, China; 4 University of Chinese Academy of Sciences, Beijing 100049, China

**Keywords:** Di-phase membrane device, organic phosphonic acid, replenishing feed section, replenishing resolving section, cobalt ion

## Abstract

A novel Di-phase membrane device with DZ272 (DDD) containing a replenishing feed section and replenishing resolving section for the purification behavior of Co(II) has been studied. The replenishing feed section was composed of feed solution and Di-isooctylphosphinic acid (DZ272) as the carrier in fossil oil, and the replenishing resolving section was composed of DZ272 as the carrier in fossil oil and HCl as the resolving agent. The effects of the voluminal ratio of the membrane solution and feed solution (O/F), pH, initial molarity of Co(II) and ionic strength in the feed solution, voluminal ratio of membrane solution and resolving agent (O/S), molarity of H_2_SO_4_ solution and DZ272 molarity in the replenishing resolving section on purification of Co(II) were considered. The benefits of DDD compared to the traditional membrane device, system stability, reuse of the membrane solution and retention of the membrane section were also studied. Experimental results indicated that the optimal purification conditions of Co(II) were obtained, as H_2_SO_4_ molarity was 2.00 mol/L, DZ272 molarity was 0.120 mol/L, O/S was 3:1 in the replenishing resolving section, O/F was 1:8 and pH was 5.20 in the replenishing feed section. The ions intensity in the replenishing feed section had no apparent effect on purification behavior of Co(II). When the initial Co(II) molarity was 3.00 × 10^−4^ mol/L, the purification percentage of Co(II) achieved 93.6% in 200 min. The kinetic equation was deduced in light of the law of mass diffusivity and interfacial chemistry.

## 1. Introduction

The removal of toxic heavy metals such as cadmium, platinum, cobalt, copper, chromium, silver, gold, mercury and zinc from aqueous environments has received considerable attention in recent years due to their toxicity and carcinogenicity, which may cause damage to various systems of the human body. They can also be readily adsorbed by marine animals and directly enter the human food chain, thus presenting a high health risk to consumers [1]. Cobalt ions are non-biodegradable toxic heavy metals and may cause dermatitis and allergic sensitization [2,3]. According to the World Health Organization, cobalt in effluents in the United States from the electroplating procedure slops is 4.1 mg/L, while that in drinking water should be less than 0.1 mg/L [4]. The major sources of cobalt contamination in water come from industrial production procedures such as aviation, petrochemical, electroplating, battery manufacturing, mining, metallic materials and many other industries. It is necessary to remove and recover these highly toxic and nonbiodegradable heavy metals in order to meet increasingly stringent environmental quality standards and promote the recycling and reuse of heavy metallic resources [5]. Traditional methods used for these purposes include adsorption, reverse osmosis, filtration, chemical precipitation, adsorbents (such as: ion exchange), biological systems, solvent extraction procedures, fluid membrane, evaporation, etc. Among these methods, sedimentation is usually undesirable because the sludge it produces must fill the land, the relevant metals cannot be recovered, the chemical cost is high, and it is a semi-continuous procedure. Biological systems are usually unstable and their dynamics are slow. Adsorbents can have high selectivity, capacity and adsorption rate, but they can only work in semi-continuous operation, in which the adsorbent must be regenerated regularly [6,7,8]. Evaporation, reverse osmosis and electrodialysis have no selectivity, while precipitation, solvent extraction and ion exchange allow for the recovery of metal ions, but they are rarely used, because the capital and operating costs are higher than the value of recycled materials [9]. Therefore, it is necessary to develop more efficient and cost-effective removal and recovery methods to overcome these difficulties.

Numerous industries had paid more and more attention to fluid membrane (FM) techniques due to the specific features in recent years. The potential benefits of FM techniques, over traditional purification methods and solid membrane techniques, are low property and running costs, low power exhaust and extractant consumption, high extraction efficiency and high extraction percentage [9,10]. FMs can carry out simultaneous extraction and resolving procedures in the same part, and they have benefits of nonequilibrium mass transfer and up-hill effect, where the solute can move from low to high molarity solution. The main type of liquid membrane system includes emulsion fluid (EF) and a membrane device (MD). The purification rates of FM, EF and MD (reverse osmosis and electrodialysis, nanofiltration) were about 70–80%, 80–85% and 70–90% (90%, 75%, 85%), respectively. The removal rates are not high [10].

Extraction technology and membrane technology are better methods to treat industrial heavy metal slops. Especially, membrane technologies have broad application prospects. Some scholars used extraction methods to dislodge the heavy metals and compounds, but the efficiency was very low, and the effective use times were less (about 70–75%) [11,12,13,14,15]. Recently, some scholars have used new liquid membrane technology to dislodge heavy metals [16]. However, due to the lack of supplementary solution, the dislodge efficiency was low (about 85%), the membrane was used less times, and the cost was high [17,18]. Some people also used a hollow fiber liquid membrane device to study the dislodging of metal molybdenum [18]. It was found that the removal rate could be increased by 10% after the supplementary liquid was used, and the use times of the membrane increased significantly [17,19]. Some people also used an ion-exchange membrane to dislodge heavy metal compounds, and the effect was significantly lower than that of membrane technology [9]. Many studies also showed that the parameter selection of liquid membrane technology is similar to that of extraction technology, but the dislodging efficiency was much higher [20]. Because the organic membrane solution is volatile, the stability of the liquid membrane and the extraction method are also affected [21]. Therefore, whether the liquid membrane technology can operate in a closed manner will be the focus of the research. The volatilization of the organic membrane solution is controlled, which can improve the removal rate and stability [18].

Some novel fluid membrane configurations have been studied in order to overcome these difficulties, such as supported emulsion fluid membrane, hollow fiber fluid membrane, etc. [5]. In previous studies, one replenishing supported liquid membrane, named the dispersion supported fluid membrane, has been studied for the purification of rare Earth metals, and the purification results were apparent [10,19,22]. In this study, a novel fluid membrane technique, named Di-phase membrane device with DZ272 (DDD), was investigated for the removal and recovery of target species from a feed solution. This is a new fluid membrane procedure, which has some benefits compared with traditional MD, previously used. The effects of various experimental parameters on the purification of Co(II) in slops were studied. The practical application of this technology can break through the bottleneck of water treatment and membrane technology. It provides scientific and theoretical support for industrial slops treatment, especially for the purification and extraction of heavy metals.

## 2. Materials and Methods

### 2.1. Theoretical Methods

**Figure 1:**
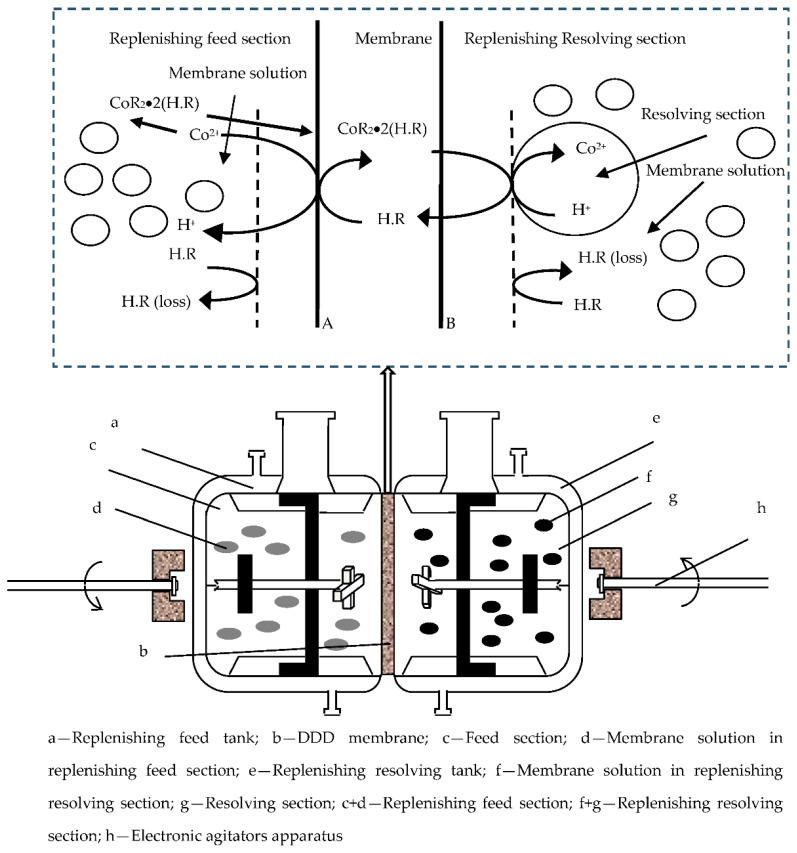
Mechanism schematic of Co(II) purification through the Di-phase membrane device.

Figure 1 is the principle of DDD, in which purification procedures and molarity changes are depicted. Specific explanations of the mechanism are given in references [20,21] below.

H.R represents the carrier within the membrane, which in this case is DZ272. CoR_2_•2(H.R) represents the organometallic compound, H^+^ is the hydrogen ion, and Co(II) is the uncomplexed Co(II) (A and B are the boundaries of the membrane section).

The DDD technology was composed of three sections: (1) the replenishing feed section concluding a metallic-ion and membrane solution. (2) the membrane section of KYNOAR impregnated with an organic solution containing DZ272 and solvent, of which the membrane serves as a uniform barrier between the replenishing feed tank and replenishing resolving tank.; (3) replenishing resolving section containing resolving solvent and membrane solution in which the metallic-ion is uncomplexed from membrane section.

The two reactions at interfaces A and B in Figure 1 are:

(1) $${\mathrm{Co}}_{f}{}^{2+}+2{\left(H.R\right)}_{2,\mathrm{org}}\overset{K_{1}}{\underset{K_{-1}}{⇄}}\mathrm{Co}R_{2}.2H.R_{\left(\mathrm{org}\right)}+2H_{f}{}^{+}$$

(2) $$\mathrm{Co}R_{2}.2H.R_{\left(\mathrm{org}\right)}+2H_{r}{}^{+}\overset{K_{2}}{\underset{K_{-2}}{⇄}}{\mathrm{Co}}_{r}{}^{2+}+2{\left(H.R\right)}_{2,\mathrm{org}}$$

where f, r and ogr stand for feed section, resolving section and organic solution, respectively. K_1_, K_−1_, K_2_, K_−2_ are the Quasi first level rate constants of the positive reaction and reverse reaction of boundaries A and B.

The final kinetic analysis is based on Equations (3)–(5):

(3) $$\frac{1}{P_{c}}=\Delta_{f}+\Delta_{o}\frac{1}{K_{1}}\frac{{\left[H^{+}\right]}^{b}}{{\left[{\left(H.R\right)}_{2}\right]}^{\frac{a+b}{2}}}$$

Δ*_f_* and Δ_0_ can be expressed as below:

(4) $$\Delta_{f}=\frac{d_{f}}{D_{f}}$$

(5) $$\Delta_{0}=\frac{d_{0}}{D_{0}}$$

where *D_f_*, *d_f_* represent diffusivity parameters and the thickness of the aqueous liquid and membrane interface, and where *D*_0_ and *d*_0_ represent the diffusivity parameters and thickness in the membrane section.

The relevance of *1/P_C_* and [H^+^]^n^ are examined to be linear at the same carrier molarity. Thus, the diffusivity constant of Co(II) in the membrane and the thickness of diffusivity layer between the replenishing feed section and membrane section can be obtained with the linear slope method. In the same way, the relevance of *1/P_C_* and [(H.R)_2_]^−2^ were examined to be linear at the same H^+^ molarity in the replenishing feed section.

### 2.2. Reagents and Devices

Di-isooctylphosphinic acid (DZ272) was used as the carrier in this work, with a density of 0.839, and purity of about 95% (Shanghai laiyashi Chemical Co., Ltd., Shanghai, China). A H_2_SO_4_ and CoSO_4_ solution was mixed as feeding solution to imitate industrial slops containing CoSO_4_. The buffer solution (HAc-NaAc) was applied for the pH adjustment (2.4–4.5) of the feeding solution, and the mixed solution of K_2_SO_4_ and KCl was applied for the monitor of the ion-density in the feeding section to imitate the original industrial slops. Sulfuric acid was selected as the resolving liquid, and the homemade fossil oil was used as the organic solvent. The mixed solution of fossil oil with DZ272 and the sulfuric acid solution were used as the supplying solution. All the reagents (except fossil oil) were of analytical grade. Chemical reagents had no special label, most of which were from a chemical company in Nanchang city, Jiangxi province (Jiangxi Yatai Chemical Co., Ltd., Nanchang, China).

Cobalt sulfate (CoSO_4_) from the simulated slops according to the actual slops of the metal mine in Jilin Province, as well as acetic acid glacial and sodium acetate anhydrous (HAc-NaAc) and 4-(2-pyridyla20) resoroin (PAR) used in the present work were of analytical grade. All chemical reagents were dissolved by deionized water. Di-isooctylphosphinic acid (DZ272) is a commercial extractant (purity >95%) and was used without any further purification. Fossil oil was washed with concentrated sulfuric acid and distilled at 180–200 °C.

All the experiments were conducted using quantities of 110 mL. The device contained two tanks that were separated by a Kynoar film as the membrane. The membrane had a porosity of 70%, refractive index of 1.71, thickness of 63 μm and pore size of 0.40 μm. The membrane effect area was 12.3 cm^2^. The JJ-1 precision-strengthened electronic agitators were made in Shanxi province, China. The UV1200s spectrophotameter was made in Shanghai, China. The water purification device was made in Zhejiang province, China.

### 2.3. Experimental Procedure

The replenishing feed section was an aqueous solution that contained Co(II) and the membrane solution. The metal solution was prepared by dissolving the required amount of Co(NO_3_)_2_.4H_2_O. The replenishing resolving section was the mixture of aqueous fluid containing H_2_SO_4_ and the membrane fluid. The membrane fluid was prepared by dissolving DZ272 in fossil oil. The KYNOAR film used as a membrane was immersed with the required amount of membrane fluid for more than 5 h in order to make the micropores filled with DZ272. The tests were carried out in a pH range of 3.3–5.6 to investigate the pH effect of replenishing the feed section on purification, the hydrogen ion molarity of the replenishing resolving section, the voluminal ratio of the membrane solution and H_2_SO_4_ and the molarity of Co(II) of the replenishing feed section. The pH of the replenishing feed section at each part of the experiment was kept constant using buffer solutions during other conditional experiments [15,23].

The Co(II) molarity was analyzed by spectrophotometric (510 nm) with developer PAR. The pH (+0.02 pH) was measured with a PHS-3C digital precision ionization meter with a combined glass electrode.

## 3. Results and Discussion

### 3.1. Stability of DDD

In order to define the stability of DDD compared with a traditional membrane device, the tendency of Co(II) molarity changes in the replenishing feed section and replenishing resolving section with time were studied under a fixed operating condition for a long time.

**Figure 2:**
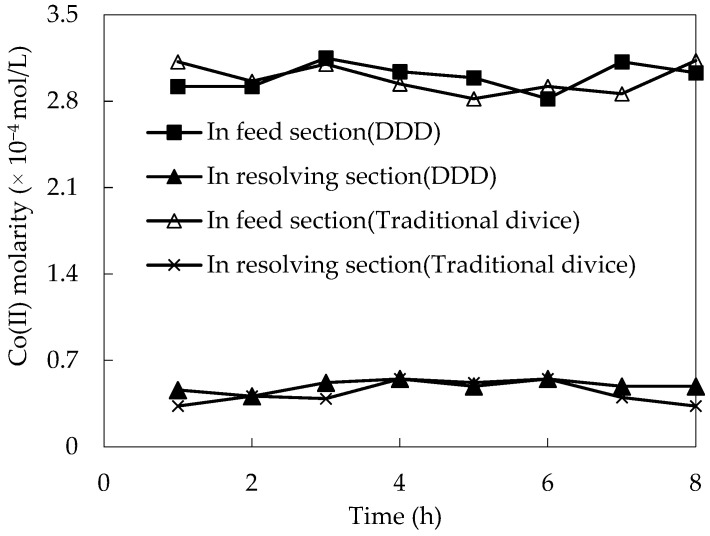
The system stability comparison of DDD and a traditional membrane device.

The assumed experimental conditions were chosen in a certain pH in the replenishing feed section, which was configured to 4.80. The initial molarity of Co(II) was 2.00 × 10^−4^ mol/L; the voluminal ratio of the membrane solution and replenishing feed solution (O/F) was 1:2 in the replenishing feed section, the voluminal ratio of the membrane solution and H_2_SO_4_ fluid (O/S) was 2:1, the molarity of H_2_SO_4_ was 1.50 mol/L, and the molarity of DZ272 was 0.110 mol/L in the replenishing resolving section. The results are shown in the Figure 2. Every 60 min, the change in Co(II) molarity was stable; thus, we took samples every 60 min in each purification test. We found that after 7.0 h, the Co(II) molarity and stability of the replenishing resolving section decreased gradually when using a traditional membrane device. Meanwhile, the Co(II) molarity in both the replenishing feed section and the replenishing resolving section remained stable when using DDD. This is because the carrier (DZ272) in a traditional membrane device gradually loses, and DDD with the replenishing section can replenish DZ272 to the membrane system [24,25]. Therefore, we can conclude that the stability of DDD is better than that of traditional membrane devices.

### 3.2. Effect of the Voluminal Ratio of the Membrane Solution and Feed Solution (O/F)

The effect of the voluminal ratio of the membrane solution and feed solution (O/F) was studied in this section. All other parameters such as pH, molarity of Co(II) in the replenishing feed section, molarity of the H_2_SO_4_ solution and the voluminal ratio of the membrane solution and H_2_SO_4_ solution (O/S) in the replenishing resolving section were kept constant at 4.8, 2.0 × 10^−4^ mol/L and 3, respectively. The effect of the voluminal ratio of the membrane solution and feed solution (O/F) in the replenishing feed section on purification of Co(II) is shown in Table 1, which indicates that the purification percentage of Co(II) decreased with the increase in the voluminal ratio from O/F 0.1 to 1.0. When the voluminal ratio of O/F was 0.1, the purification percentage of Co(II) was 82.3%. Considering saving chemical agents as well as increasing the purification percentage, we chose 0.1 mol/L as the optimal voluminal ratio for the following experiments.

### 3.3. Effect of pH on the Replenishing Feed Section

**Figure 3:**
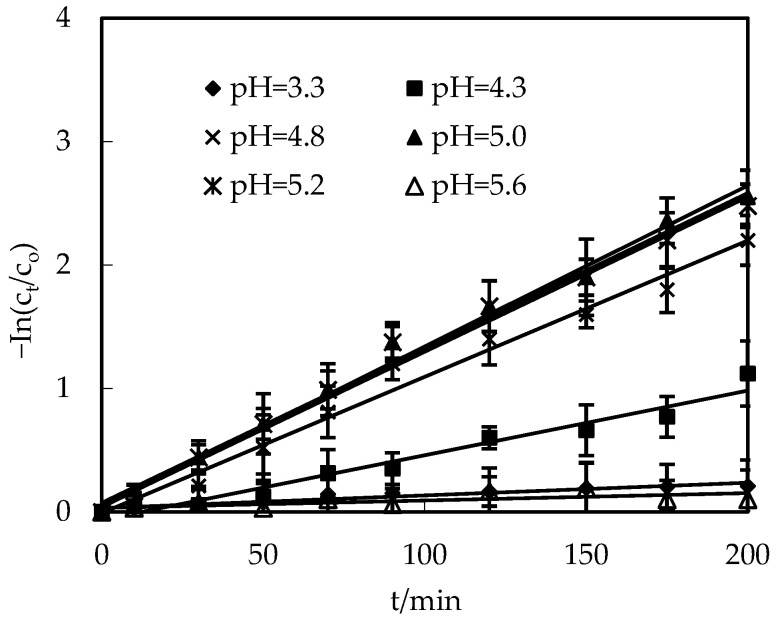
The pH effect in the replenishing feed section on Co(II) purification.

The effect of pH on the purification of Co(II) was explored in pH values ranging from 3.3 to 5.6, which was configured with an acetate sodium/acetic acid buffer fluid. The O/F was configured to 0.125. The molarity of Co(II) in the replenishing feed section was 2.0 × 10^−4^ mol/L. The molarity of H_2_SO_4_ in the replenishing resolving section was 2.0 mol/L, and the O/S was 3. The results are presented in Figure 3. The Co(II) percentage increased as the pH of the feed section increased from 3.3 to 5.0, and a max percentage was determined at pH 5.0. Above pH 5.0 in the feed solution, the Co(II) percentage decreased. This is similar to the influence of pH on the distribution coefficient of the extraction procedure [16]. It is large because the purification procedure is mainly governed by the mass transfer driving force caused by the distribution equilibrium, when the renewal effect of the liquid membrane and the diffusivity mobility of Co(II) ions are determined under specific experimental conditions [2,23].

### 3.4. Effect of Initial Molarity of Co(II) on the Replenishing Resolving Section

**Figure 4:**
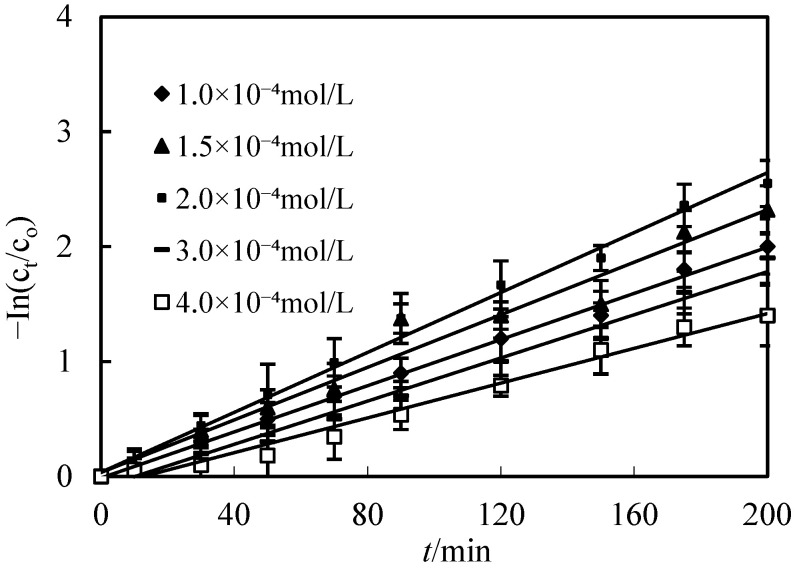
Effect of initial molarities of Co(II) on the purification of Co(II).

The effect of Co(II) molarity on percentage and the purification factor Co(II) was studied in the range of 1.0 × 10^−4^ to 4.0 × 10^−4^ mol/L. The pH of the replenishing feed section was configured to 5.2. The O/F was configured to 0.125. The O/S was configured to 3, and H_2_SO_4_ was configured to 2.0 mol/L in the replenishing resolving section. The results obtained are presented in Figure 4. With the increase in Co(II) molarity in the replenishing feed section from 1.0 × 10^−4^ to 2.0 × 10^−4^ mol/L, the percentage of Co(II) increased and then decreased while the initial molarity of Co(II) increased in the replenishing feed section. Within this molarity range of Co(II) in the replenishing feed section, the availability of Co(II) at the feed–membrane interface increased with increasing Co(II) molarity. However, when the Co(II) molarity in the feed solution became higher in comparison to the DZ272 molarity in the membrane section, the percentage of Co(II) decreased. This indicates that the number of moles translated through the membrane per unit area of the membrane per unit time are determined when the molarity of DZ272 as well as the effect area of the membrane and time are determined.

### 3.5. Effect of the Voluminal Ratio of the Membrane Solution and H_2_SO_4_ Solution (O/S)

**Figure 5:**
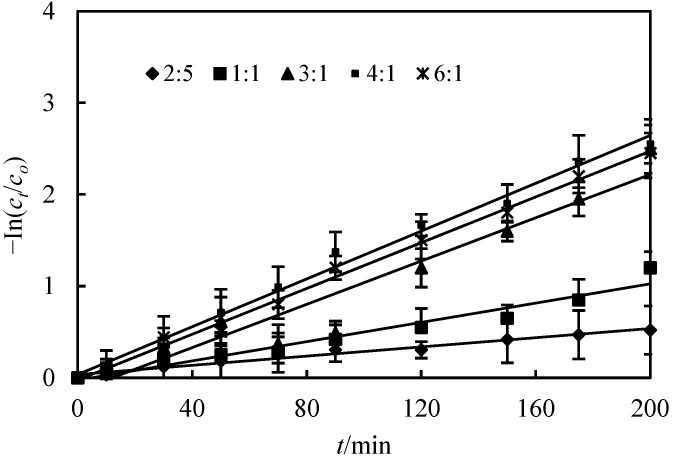
Effect of the voluminal ratio of the membrane solution and H_2_SO_4_ solution on purification of Co(II).

The effect of the voluminal ratio of the membrane solution and H_2_SO_4_ solution (O/S) in the replenishing resolving section on the purification percentage of Co(II) is shown in Figure 5. It makes sure that the percentage of Co(II) increased with an increase in the voluminal ratio. When the voluminal ratio of O/S increased, the droplets of the resolving fluid spread in the membrane section, which apparently increased [4,7]. In this way, the membrane section and replenishing resolving section provide an extra resolving surface and promote a renewal rate of the liquid membrane, which leads to an extremely resolving rate for the replenishing feed section from the membrane section and to an extension of the life of the liquid membrane. Therefore, it enhances the purification percentage of Co(II).

### 3.6. Effect of the Molarity of H_2_SO_4_ in the Replenishing Resolving Section

**Figure 6:**
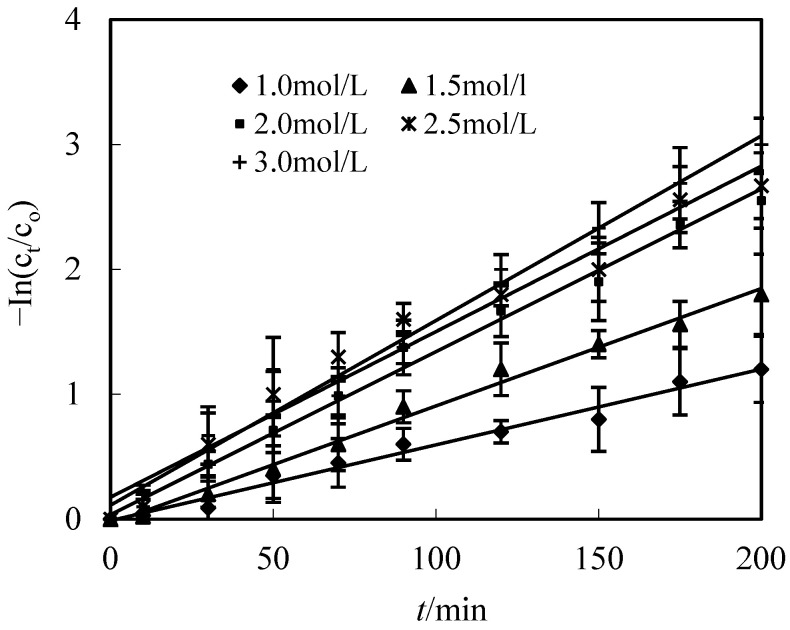
Effect of molarities of H_2_SO_4_ in replenishing resolving section on purification of Co(II).

The resolving reaction on the membrane-resolving side plays a crucial role in the purification of metallic ions from the replenishing feed section to the replenishing resolving section. Thus, the effect of the molarity of H_2_SO_4_ was studied in this paper. All other parameters such as pH, molarity of Co(II), O/F of the replenishing feed section and O/S were configured to 0.125, 5.2, 2.0 × 10^−4^ mol/L and 3, respectively. Figure 6 shows the effect of purification efficiency of Co(II) in different molarities of H_2_SO_4_, which indicates that the Co(II) percentage increased with the increase in acid molarity. Further increases in the molarity of H_2_SO_4_ from 2.0 to 3.0 mol/L had no significant effect on the percentage of Co(II), because the number of the complexes of Co(II) and DZ272, of which there is purification through the membrane per unit area of the membrane per unit time, are determined when the molarity of DZ272, the molarity of Co(II) in the replenishing feed section, and the effective area of the membrane and time are defined. Thus, the molarity of H_2_SO_4_ in the replenishing resolving section was 2.0 mol/L in the best condition.

### 3.7. Effect of Ionic Intensity in the Replenishing Feed Section

The effect of ionic intensity in the replenishing feed section on percentage of Co(II) was studied in this paper. The results are shown in Table 2. They indicate that the ionic intensity had no effect on the purification of Co(II), due to H_2_SO_4_ being contained by the membrane fluid, which led to the ionic intensity of the replenishing resolving section, which can be ignored. Therefore, when the molarity of ions in the replenishing feed section are low, the ionic intensity of the two sections should be neglected. Compared with the other liquid membrane technologies, the operating conditions are further simplified.

### 3.8. Reuse of the Membrane Solution

Reuse of the membrane solution was studied under optimal conditions. From Table 3, we know that the membrane solution in DDD can be reused many times before re-extraction with the strong acid after every experiment. The membrane solution in DDD can be reused many times, and the tendency of the purification percentage changes was stable with DDD in seven experiments. After seven experiments, the Co(II) purification percentage gradually decreased.

### 3.9. Reuse of the Membrane Sheet

Reuse of the membrane sheet was studied under the best conditions. The membrane solution was reused for only seven tests, and we changed the new membrane sheet or membrane fluid after purification with a strong acid reverse extraction in the seventh test. The results are shown in Table 4. The membrane sheet of DDD with the replenishing resolving section can be reused many times, and the tendency of the purification percentage changes was stable with DDD in nine experiments, but under the condition of the membrane sheet of the traditional membrane device without the replenishing section, the percentage change trend of the purification was unstable, and the purification percentage decreased gradually after four experiments. In this study, we can also conclude that the stability of DDD is better than that of traditional membrane devices (traditional liquid membrane, emulsion membrane and ion exchange membrane).

### 3.10. Retention in the Membrane Section

**Figure 7:**
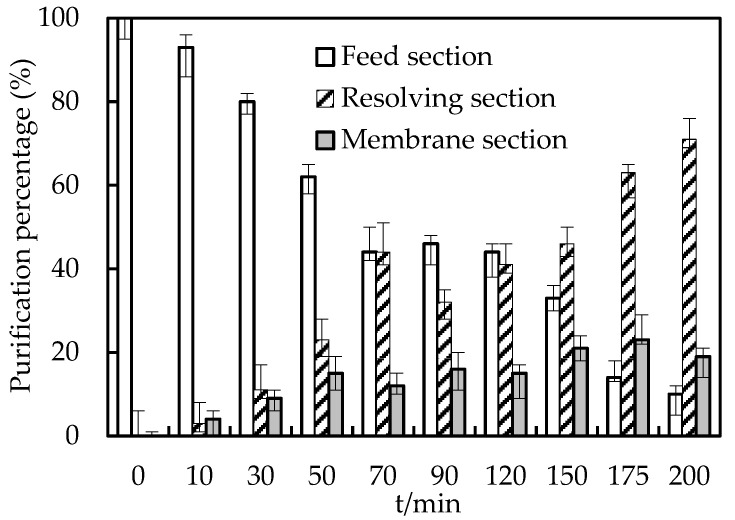
Retention in the membrane section and effect of resolving.

Retention in the membrane section and the resolving effect were studied under the best conditions. The pH was configured to 5.20, O/F was configured to 0.10, initial molarity of Co(II) was 2.00 × 10^−4^ mol/L in the replenishing feed section, O/S was configured to 4.00, and the molarity of the H_2_SO_4_ solution was also configured to 2.00 mol/L in the replenishing resolving section. According to the molarity of both the replenishing feed section and the resolving section, the molarity of Co(II) in the membrane section can be obtained, and then, the effect of resolving in the replenishing resolving section and retention phenomenon of the membrane section can be obtained. The results are shown in Figure 7.

## 4. Kinetic Analysis

**Figure 8:**
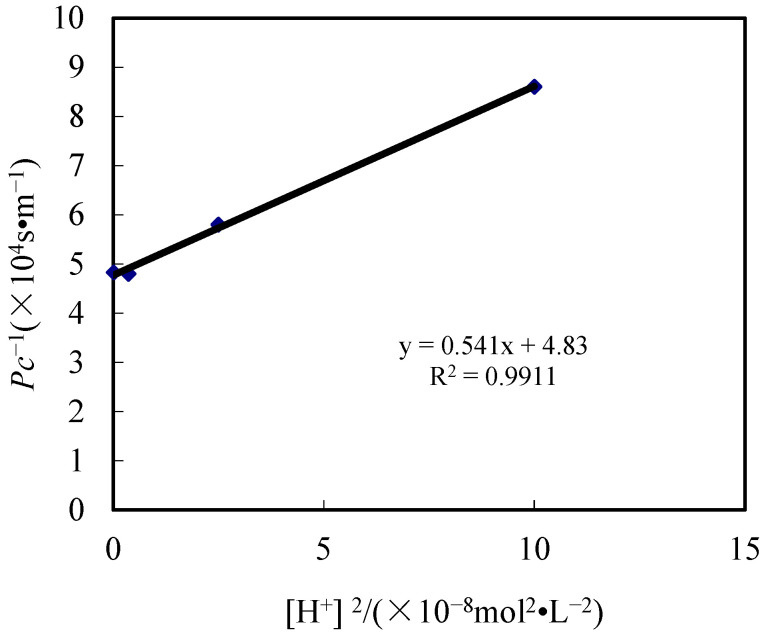
Dynamic relationship between the experimental results and theory.

According to the data of the pH effect in the replenishing feed section, the relevance between [H^+^]^2^ and *1/P_C_* was revealed. When determining the molar concentration of the mobile carrier, *P_C_* was also determined. The results are shown in Figure 8.

They show that there was a linear relevance of 1/P_C_ and [H^+^]^2^ at a certain pH value. The R^2^ was 0.9911, which is in good agreement with the theory from Equattion 1. The slope and intercept were 5.41 × 10^12^ and 4.83 × 10^4^. The thickness of the diffusivity layer *d_f_*, which was calculated by using the diffusivity constant of Co(II) in the aqueous fluid, which is 6.92 × 10^−10^ m^2^/s [9,26,27,28], was 1.08 × 10^−4^ m. *K*_1_, which can be determined by the extraction test, was 2.7 × 10^−8^. Then, *D_o_* of the diffusivity constant in the membrane was 1.31 × 10^−7^ m^2^/s according to Equations (4) and (5). The new kinetic equation was determined by *d_f_* and *D_o_* in the DDD system. It can be represented as

(6) $$P_{C}=\frac{1}{4.83\times 10^{4}+5.41\times 10^{12}{\left[H^{+}\right]}^{2}}$$

## 5. Conclusions

The purification of Co(II) in slops with DDD using DZ272 as a mobile carrier were studied. The following conclusions were drawn from the above studies.

(1) The optimal conditions for purification of Co(II) were that the molarity of sulfuric acid was 2.0 mol/L, O/F was 1:8, DZ272 molarity was 0.150 mol/L and O/S was 3:1 in the replenishing resolving section, initial molarity of Co(II) was 3.0 × 10^−4^ mol/L, and pH was 5.0 in the replenishing feed section. When the purification time was 200 min, the purification percentage was 93.6% in optimal conditions.

(2) Throughout the experiment, a model was established to describe the reaction and purification of metal ions in the DDD. The new kinetic equation was deduced. The diffusivity coefficient in the membrane section and the thickness of the diffusivity layer in the replenishing feed section were obtained by the linear slope method. They were 1.31 × 10^−7^ m^2^/s and 1.08 × 10^−4^ m, respectively.

(3) In the DDD, as a result, a large amount of membrane fluid was used, which can replenish the losing carrier (DZ272) in the membrane device. Consequently, while increasing the purification percentage of Co(II), it also improved the stability of the membrane and extended the life of the membrane.

Based on the above research, we can fully carry out the practical engineering of industrial water treatment. Next, our research will focus on practical industrial applications. The practical application of this technology can break through the obstruction of water treatment and membrane technology. It will provide scientific and theoretical support for industrial slops treatment, especially for the purification and extraction of heavy metals.

## Figures and Tables

**Table 1:** Effect of voluminal ratio of the membrane solution and feed solution on purification of Co(II).

Time (min)	Purification Percentage (%)				
	0	0.1	0.25	0.5	1.0
0	0	0	0	0	0
30	23.3 ± 2.1	32.7 ± 1.9	29.8 ± 1.1	27.8 ± 2.8	19.7 ± 1.2
50	39.8 ± 1.7	54.6 ± 3.6	47.1 ± 1.9	46.1 ± 4.6	31.8 ± 2.3
140	515 ± 3.3	63.7 ± 2.5	52.3 ± 3.9	51.2 ± 1.5	49.4 ± 1.8
170	61.9 ± 3.2	76.2 ± 4.3	62.4 ± 4.2	65.3 ± 5.1	59.5 ± 4.2
200	77.3 ± 2.4	82.3 ± 3.1	78.3 ± 3.5	74.9 ± 4.9	67.0 ± 2.1

Note: The values in the table are the average values ± standard deviation.

**Table 2:** Ionic intensity effect on the purification of Co(II).

Time (min)	Purification Percentage (%)				
	0.5	1.1	1.75	2.0	2.5
0	0	0	0	0	0
30	29.2 ± 2.1	32.7 ± 1.9	33.1 ± 1.9	32.6 ± 4.0	29.8 ± 0.4
50	40.8 ± 1.6	54.6 ± 3.6	47.1 ± 3.9	46.1 ± 4.6	35.2 ± 3.0
140	59.9 ± 3.1	63.7 ± 2.5	53.4 ± 1.8	51.2 ± 1.5	47.4 ± 3.9
170	70.5 ± 3.4	76.2 ± 4.3	69.8 ± 2.5	65.3 ± 5.1	60.2 ± 1.2
200	82.3 ± 1.6	82.3 ± 3.1	81.3 ± 6.3	84.9 ± 4.9	79.8 ± 2.5

Note: The values in the table are the average values ± standard deviation.

**Table 3:** Reuse of the membrane solution.

Time (min)		Purification Percentage (%)						
	1	2	3	4	5	6	7	8
0	0	0	0	0	0	0	0	0
30	33.4	35.7	39.2	32.6	36.7	39.2	39.2	31.1
50	49.8	47.7	54.0	50.2	52.1	51.7	54.0	43.7
140	63.9	69.0	63.2	60.7	64.6	62.2	60.7	59.2
170	78.5	79.8	74.5	77.8	75.6	79.1	77.7	71.3
200	89.3 ± 1.9	91.0 ± 4.8	89.4 ± 5.8	91.2 ± 2.7	89.3 ± 4.7	92.3 ± 4.5	90.2 ± 4.9	85.1 ± 5.3

Note: The values in the table are the average values ± standard deviation.

**Table 4:** Reuse of the membrane sheet.

Technology Type		Purification Percentage (%)						
	1	2	3	4	5	6	7	8
DDD	91.2 ± 3.8	93.1 ± 5.8	92.7 ± 6.1	90.6 ± 4.2	90.4 ± 2.8	91.3 ± 3.9	88.7 ± 5.2	85.4 ± 3.7
Traditional liquid membrane	83.4	85.2	79.5	82.7	76.3	75.8	69.7	61.7
Emulsion membrane	79.9	77.2	74.4	70.6	62.3	61.9	44.7	43.1
Ion exchange membrane	81.5	81.8	79.5	61.4	69.3	52.9	40.9	35.4

Note: The values in the table are the average values ± standard deviation.

## Data Availability

This study does not report any data.
